# Supplementary material for: Are carbon emissions trading and green financial instruments synergistic? -Comprehensive quantitative research based on content analysis
Source: PLoS One. 2024 Mar 7;19(3):e0298601. doi: 10.1371/journal.pone.0298601 (PMC10919601; doi:10.1371/journal.pone.0298601)
Supplement: S1 Data — (ZIP) [file pone.0298601.s001.zip › Data/地级市Z_F工作报告.zip!/╕ú╜¿/╚2├≈╩╨/└┤╫╘╣1⁄2╓┌║┼í╛┬φ┐╦╩2╛▌═°í┐_2018.docx]

各位代表：

　　现在，我代表三明市人民政府向大会作政府工作报告，请予审议，并请市政协各位委员和其他列席人员提出意见。

　　一、2017年工作回顾

　　2017年是本届政府的开局之年，全市各级政府各部门认真学习贯彻党的十九大精神，以习近平新时代中国特色社会主义思想为指导，全面贯彻落实党中央、国务院和省委、省政府以及市第九次党代会决策部署，全力稳增长、促改革、调结构、惠民生、防风险，经济社会呈现稳中向好、稳中有进、稳中趋升的态势。初步统计，全市生产总值2136.06亿元，增长7.9%；规模以上工业增加值847.18亿元，增长8.1%；地方一般公共预算收入100.76亿元，增长6.4%；固定资产投资2498.50亿元，增长16.7%；外贸出口146.9亿元，增长12.1%；实际利用外商直接投资1.84亿美元，增长7.9%；社会消费品零售总额533.43亿元，增长11%；城镇居民人均可支配收入32261元，增长8.7%；农村居民人均可支配收入15212元，增长9.3%；居民消费价格总水平上涨0.7%。一些重点领域和项目取得突破：

　　——重点项目有新亮点。中国化工集团双轮化机智能注塑机、省电子信息集团智能化车载模组、三钢物联云商、厦钨三元正极材料、致格锂电池、泰达铝硅合金、翔丰华石墨负极材料二期、东莹化工新型环保制冷剂、明一国际高新科技园等一批产业项目开工或投产。厦沙高速建成通车，兴泉铁路、浦梅铁路、莆炎高速三明段全线开工，三明沙县机场旅客运输量突破20万人次。闽江流域山水林田湖草生态保护修复工程试点项目获批。

　　——改革创新有新拓展。我市医改工作在中央深改组第33次会议上得到肯定，被确定为公立医院综合改革首批国家级示范城市，我市和尤溪县受到国务院表扬激励；林改得到中央肯定，全国、全省林业改革现场会在我市召开；我市被评为全国生态保护与建设典型示范区。

　　——难题攻坚有新突破。开展精准脱贫攻坚行动，预计全年脱贫2.8万人；开展信贷风险化解攻坚行动，全年化解不良贷款96.9亿元，信贷不良率从年度最高的5.42%下降到2.7%，超额完成省委省政府下达的目标任务；开展市区和谐征迁百日攻坚行动，破解了一批困扰多年的征迁难题，累计征收土地2770亩；开展“城市双修”百日攻坚行动，20个示范项目取得良好成效。

　　——民生事业有新成效。25项省市为民办实事项目全面完成，万寿岩荣获国家考古遗址公园，高考成绩位居全省前列，全省基础教育现场会在我市召开，我市运动员在第十三届全运会上获得5金2银4铜的历史最好成绩。

　　——城市面貌有新形象。三明蝉联全国文明城市和全国综治“长安杯”，荣获全国“城市双修”试点城市；沙县首次荣膺全国文明城市称号，泰宁县荣获国家生态文明建设示范县。

　　过去一年的主要工作和成效是：

　　（一）产业转型升级步伐加快。供给侧结构性改革扎实推进，认真落实“三去一降一补”，全市关闭退出煤矿11处，压减煤炭产能61万吨；实现商品房销售275.95万平方米，库存去化周期降至13个月；开展打击金融领域恶意逃废债、公职人员不良贷款清理、涉金融案件执行等三个攻坚行动，全市金融机构贷款余额1313.42亿元、增长3.8%；出台稳定工业增长、扶持高成长性企业发展等系列政策，帮助企业降低各类成本15亿元；实施30个补短板工程包，完成投资118.5亿元，围绕教育、医疗卫生、养老和城乡民生基础设施四个领域策划实施补短板项目2037个、总投资1738亿元。传统产业提质增效，完成技改投资948.2亿元，冶金及压延、汽车及机械装备、林产加工、纺织四个传统产业实现产值2258亿元、增长16%。战略性新兴产业加快发展，制定稀土、氟化工、石墨（烯）、生物医药“一业一策”扶持政策，设立氟化工产业技术研究院、石墨（烯）产业技术研发平台、新能源材料产业技术研究院等一批科技研发平台，战略性新兴产业实现产值650亿元、增长20.3%。现代服务业提速发展，实施100个服务业重点项目，开展三明“深呼吸”旅游联盟、三明南平“大武夷”旅游联盟协作，新增大田、宁化2个国家级电子商务进农村示范县，尤溪县被认定为全国休闲农业和乡村旅游示范县，全市服务业增加值增长11.1%。品牌农业、智慧农业、生态农业加快发展，三明农特产品十大龙头品牌成效明显，新增“三品一标”农产品68个，289家市级农业龙头企业产值373.5亿元。

　　（二）“五个一批”项目接续发展。组织开展“五比五晒”项目竞赛活动，实行“一月一协调、一季一督查、一季一通报”和正向激励工作机制，全市列入省级“五个一批”项目管理库2975个、总投资8500.3亿元，其中2017年新入库项目2125个、总投资3815.5亿元。加快推进464个省行动计划重大项目和185个省级重点项目建设，130个项目开（竣）工。建宁、将乐、宁化分别获得省上“五个一批”项目正向激励奖励。完善招商引资机制，成立7个招商项目工作组，强化市县联合招商，推动“一把手”招商，全年共签约亿元以上项目433个、总投资1297.5亿元，与三钢集团签订17个、总投资85亿元的产业转型升级项目。

　　（三）重点领域改革不断深化。医改深入推进，完成以总医院为载体的紧密型医联体组建工作，尤溪、将乐“全民健康四级共保”试点走在全市前列。林改成效不断提升，创新推出“福林贷”普惠制林业金融产品，累计林权抵押贷款112.1亿元、约占全省的一半，率先在全省核发林地经营权证，国有林场改革全面完成。金融改革加快推进，发放园区企业资产按揭贷款23.74亿元，开展农村林、地、房“三权”抵押贷款业务，累计为农户贷款15亿元，在永安五一村开展“福田贷”试点。生态文明改革稳步实施，重点生态区位商品林赎买、生态执法改革有序开展，“河长制”实现四级河（段）长全覆盖，将乐县“四水”模式受到省上肯定。出台国有企业改革发展实施意见，启动市属国有企业资产重组，福建一建集团晋升建筑工程施工总承包特级资质。推进基础教育办学体制改革，试行“总校制”，建立“1+4”正向激励机制，实施三明医学科技职业学院托管职教园改革。抓好全国小微企业创业创新基地城市示范工作，新增各类市场主体3.24万户、增长12.1%。加大对外开放力度，明港澳台侨交流合作持续深化，复制推广福建自贸区创新成果60项，“陆地港”外贸通关更加便利，成功举办第十三届林博会、闽浙赣皖协作区第十九次市长联席会。

　　（四）城乡区域发展协调推进。突出推进市区经济发展，195个市区经济重点项目完成投资126.59亿元，九个专业特色工业园区加快发展，策划107个、总投资95.5亿元的“城市双修”试点项目全面启动，国道205线市区过境线至三明南站连接线、列东污水处理厂搬迁扩建、市区公共自行车道、新市路道路提升和交通改善工程等一批项目顺利实施。生态新城加快建设，北大附属实验学校、欢乐大世界水上乐园等一批项目建成投入使用，清华启迪医养、铁路冷链物流等项目有序推进。县域经济持续壮大，永安保持全省县域经济实力“十强”县（市），宁化、建宁入选全省县域经济发展“十佳”县，5个省级扶贫开发工作重点县加快发展，永安、大田新型城镇化试点稳步推进，明溪药谷小镇、永安石墨小镇等省级特色小镇建设进展顺利。全面启动生态环境综合治理攻坚行动，中央环保督察反馈问题有效整改，市区空气质量状况“优、良”天数比例达98.9%，10个县（市）环境空气质量均达到或优于国家二级标准，县级以上集中式饮用水源地水质达标率100%，辖区三条主要水系国（省）控断面水质达标率96.4%。

　　（五）社会各项事业持续进步。社会保障体系不断完善，全市新增城镇就业1.85万人，城镇登记失业率2.35%；完成全民参保登记任务，大幅度提高农村居民最低生活保障标准，市区和永安、沙县、大田率先实现城乡低保标准一体化；保障性安居工程新开工4361套，基本建成4136套。国家扶贫改革试验区加快建设，扶贫资金量化折股、金融小额信贷扶贫、造福工程易地搬迁等工作深入开展，全市完成造福工程2623户、10972人，累计发放扶贫小额信贷4.15亿元，在梅列、三元两区开展“城市困难家庭精准帮扶试点”。文教卫体事业加快发展，推进三明厦门教育山海协作，新增中小学学位6225个、幼儿园学位9310个，普惠性学前教育资源覆盖面达76%；与省上高水平医院建立协作关系，启动开展家庭医生签约服务，市医养结合服务中心投入运营，荣获全国创建幸福家庭活动示范市；拓展国家公共文化服务体系示范区建设成果，宁化、清流、明溪列入国家级客家文化（闽西）生态保护实验区，成功举办红色文化高端论坛；群众体育广泛开展，竞技体育长足发展，泰宁世界华人马拉松赛、大田全国健美操冠军赛等赛事品牌影响力不断提升。深化全国文明城市创建，贯彻执行《三明市城市市容和环境卫生管理条例》，开展市区“二手车”占道经营、“僵尸车”清理等专项整治，城市人居环境有效改善。社会保持安定稳定，圆满完成厦门金砖会晤、党的十九大等安保维稳工作，安全生产、食品药品安全监管工作不断加强，荣获全国青少年普法教育示范区，全市综治“三率”居全省前列。老龄、工会、青少年、妇儿、残疾人工作持续推进，统计、科普、民族宗教、档案、地方志、水文、气象、防震减灾、库区移民、对口援疆工作扎实开展。双拥共建和人民防空、国防后备力量建设深入推进。

　　（六）政府自身建设得到加强。自觉接受人大法律监督和工作监督、政协民主监督，高度重视、认真办理人大代表建议和政协提案，支持民主党派、工商联和无党派人士参政议政。坚持依法行政，制定《三明市人民政府拟定法规草案和制定规章程序规定》《三明市红色文化遗址保护管理办法》《三明市市区文明行为促进办法》《三明市建筑工程施工扬尘防治管理办法》等四部政府规章，全面建立政府法律顾问制度。深化“千名干部服务千家企业”活动，帮助企业争取政策1500多项、协调资金16多亿元。大力弘扬“马上就办、真抓实干”精神，推行便民服务窗口周末无休和审批服务窗口周末预约无休工作机制，市行政服务中心完成搬迁扩容，大田县行政服务中心获评全国行政服务大厅“综合十佳”。深化“放管服”改革，推进行政职能事业单位试点改革，全面实施“多证合一”，梳理“一趟不用跑”和“最多跑一趟”事项1092项，公布实施市直部门权责清单，创新机关效能“三色督查单”工作机制，行政效率进一步提高。深入推进“两学一做”学习教育常态化制度化，严格落实中央八项规定精神，驰而不息纠正“四风”，强化行政监察和审计监督，严肃查处违纪违法案件，廉洁政府建设取得新成效。

　　各位代表，过去一年成绩来之不易，这是省委省政府和市委正确领导的结果，是全市人民团结奋斗的结果，是社会各界大力支持的结果。在此，我代表市人民政府，向为三明建设发展付出辛勤劳动的全市人民，向给予政府工作有力支持与有效监督的人大代表和政协委员、各民主党派、工商联、各人民团体、无党派人士、离退休老同志及社会各界人士，向积极发挥作用的中央和省属驻明单位、驻明部队、武警官兵、政法干警，向关心支持三明发展的港澳台同胞、海外侨胞、国际友人，表示衷心的感谢！

　　在看到成绩的同时，我们也清醒地认识到发展还面临不少困难和问题，主要有：完成年度任务有差距，部分指标未能达到年初预定目标；产业竞争力不强，龙头缺乏、链条较短，自主创新能力偏弱；部分项目质量不高，大项目特别是重大生产性项目不足；城乡发展不平衡不充分问题依然存在，规划建设和管理水平有待提升，生态保护还需加强；民生工作还有不少不如人意的地方，教育、医疗卫生、养老和城乡基础设施等领域存在短板；有的干部责任意识、担当精神还有待加强。对此，我们将认真对待，采取更加有力的措施加以解决。

　　二、2018年工作安排

　　今年是全面贯彻党的十九大精神的开局之年，是改革开放40周年，是决胜全面建成小康社会、实施“十三五”规划承上启下的关键一年。政府工作总体要求是：全面贯彻党的十九大精神，以习近平新时代中国特色社会主义思想为指导，坚持稳中求进工作总基调，坚持新发展理念，紧扣社会主要矛盾变化，按照高质量发展的要求，坚持以供给侧结构性改革为主线，贯彻落实省委省政府和市第九次党代会决策部署，统筹稳增长、促改革、调结构、惠民生、防风险各项工作，突出发展实体经济，全力打好防范化解重大风险、精准脱贫、污染防治攻坚战，促进经济社会持续健康发展。今年发展的主要预期目标是：地区生产总值增长8.5%；地方一般公共预算收入增长6.5%；固定资产投资增长15%；外贸出口增长3%；实际利用外商直接投资增长6%；社会消费品零售总额增长11%；居民消费价格涨幅3%左右；城镇登记失业率控制在4%以内；城镇居民人均可支配收入增长8.5%，农村居民人均可支配收入增长9%；完成单位生产总值能耗降低和主要污染物减排年度任务。围绕上述目标，重点抓好六个方面工作：

　　（一）突出实体经济发展，打造三明经济升级版。实体经济是三明发展的本钱，三明振兴要靠实体经济。要深化供给侧结构性改革，着力“破”“立”“降”，加快构建现代化经济体系，推动质量变革、效率变革、动力变革，促进实体经济壮筋骨、上台阶。

　　千方百计扶持实体经济健康发展。突出我市一二三产特色产业，强化“一业一策”“一企一策”，分类制定和完善扶持政策措施。高度重视民营经济，大力扶持民营企业发展，深入开展“千名干部服务千家企业”活动，落实企业减轻负担各项政策措施，扎实开展“手拉手”活动。积极盘活园区存量土地、厂房设备，加快“僵尸企业”、低效能企业处置工作，实现工业用地二次开发、二次招商。坚决打好防控金融风险攻坚战，完善信贷风险监测预警和应急处置机制，有效压降不良贷款，坚决打击违法违规金融活动，营造良好的金融生态；强化金融服务实体经济，大力推进园区企业资产按揭贷款，加大中小微企业信贷支持力度，确保全年新增信贷投放130亿元以上。

　　实施新一轮技改提升行动计划。突出冶金及压延、汽车及机械装备、林产加工、纺织四个传统产业，加大新工艺、新技术、新产品研发和推广力度，实施市级重点技术改造项目300个、完成投资200亿元以上。重点支持三钢、海西重汽等21家省级龙头企业做大做强主业，全力抓好三钢闽光物联云商、德坤高档面料织染加工、双轮化机智能注塑机研发制造基地等150个产业链项目建设，力争四个传统产业实现产值2500亿元、增长10%以上。继续淘汰一批落后产能，坚决防止已关闭企业“死灰复燃”。

　　实施战略性新兴产业倍增计划。大力发展新材料、新能源、节能环保和生物医药产业，坚定不移推动资源往产业走，重点培育壮大稀土、氟化工、石墨（烯）、生物医药四个资源型战略性新兴产业，落实“一个产业、一个专业园区、一套扶持政策”机制，加快推进厦钨稀土产业链、鼎丰科技特种等静压石墨研发及产业化、三农含氟新材料及配套中间体、汇天药业增资扩产等重大项目建设，力争战略性新兴产业实现产值780亿元、增长20%以上。

　　实施现代服务业创新发展行动计划。继续实施100个现代服务业重点项目，大力发展商贸物流、休闲旅游、健康养老三大主导产业，新增限上商贸企业50家以上；突出全域旅游建设，支持泰宁创建国家旅游度假区，大力发展乡村休闲游、特色民宿游，打造一批星级休闲集镇和特色村，力争全市游客接待量增长20%以上；推动养老与旅游相结合，打造一批健康养生和养老服务示范基地。积极培育电子商务、创意设计、文化体育三大先导产业，着力发展金融、服务外包、科技服务三大配套产业，培育协同制造、个性化定制、工业云等新业态，加快电子商务进农村示范县建设，推动生产性服务业向专业化和价值链高端延伸。坚持“房子是用来住的、不是用来炒的”定位，探索租购并举的住房制度，完善房地产市场调控长效机制，促进房地产市场平稳健康发展。

　　实施现代农业提升发展计划。严格落实粮食安全省长责任制，全市粮食播种面积稳定在320万亩以上。持续推进高优粮食、绿色林业、精致园艺、生态养殖、现代烟草等5大特色产业、20条特色农产品产业链发展，深入实施“1363”工程，新增设施农业面积1500亩以上，持续打造三明农特产品十大龙头品牌，着力创建农产品质量安全市。发展多种形式适度规模经营，大力培育家庭农（林）场、专业大户、农民合作社、农业产业化龙头企业等新型经营主体。推进一二三产融合发展，健全农业社会化服务体系，大力发展休闲农业和农产品精深加工，积极发展“互联网+农业”，支持建宁国家农村产业融合发展试点示范县建设。

　　实施创新驱动战略。加快三明国家高新区建设，鼓励企业自主创新，推进机械科学研究总院海西分院、氟化工产业技术研究院、新能源材料产业技术研究院、石墨（烯）产业技术研究院、中国纺织科学研究院福建（永安）分院、中国医药工业研究总院三明分院等六大产业研发平台建设，新增省级以上各类技术创新平台10家以上。深化小微企业创业创新基地城市示范工作，实施“双高”培育工程，重点培育一批省级高成长性企业，新增高新技术企业、科技小巨人领军企业、创新型企业、知识产权优势企业50家，规模以上高新技术产业增加值突破160亿元。落实“海纳百川”高端人才集聚计划，推广“二元制”、现代学徒制育人模式，培养和引进一批创业型领军人才、实用型技术技能人才。

　　（二）深化“五比五晒”项目竞赛，积极扩大有效投资。项目是推进各项工作的抓手，没有项目一切发展都是空谈。要持续强化项目建设，把重点放在实体经济项目上，以实实在在的举措谋划和推进项目工作，用一个个实实在在的项目促发展、惠民生。

　　强化“五个一批”项目机制。继续开展“五比五晒”项目竞赛活动，完善考评奖励机制，提升“五个一批”项目建设成效。突出项目谋划，围绕重大基础设施、传统产业转型、高新技术产业发展和现有企业增资扩产，策划生成一批战略型、税源型、生态型大项目好项目。突出开竣工环节，对“五个一批”项目实行挂图作战，按照时间节点促开工、促在建、促投产，每季度集中开竣工一批项目。突出服务保障，完善“一月一协调、一季一督查、一季一通报”机制和重大项目挂牌服务机制，并联推进重点项目要素配套、人才配套、产业配套，创造有利于项目落地、建设、增投的良好环境。规范运用PPP模式，鼓励社会资本参与公共服务、生态建设、基础设施等重点领域项目建设。

　　推进重大基础设施项目。加快浦梅铁路建宁至冠豸山段、兴泉铁路和莆炎高速公路三明段建设，建成南三龙铁路，力争开工武夷新区至沙县高速公路三明段、闽江沙溪口至三明台江航道整治工程，续建及新开工国省干线100公里，深化三明岩前至明溪胡坊等3条高速公路联络线、三明中心城市轨道交通、厦门至南昌高铁三明段等项目前期；实施安全生态水系、防洪工程、水库工程等重大水利项目，加快沙县、建宁省级综合治水试验县建设，建成兴头水库、金溪三期防洪工程；抓好农村电网改造升级、海西天然气管网三明段、泰宁池潭水电厂扩建等能源项目建设，继续推进三明核电、抽水蓄能发电等重大项目前期。

　　强化招商引资实效。整合招商资源，推进市县联合招商和“一把手”招商，充分发挥市政府招商顾问作用，深化明商“回归工程”，用好“6·18”“9·8”等招商平台，办好第十四届林博会，力争全年签约亿元以上项目总投资达1400亿元。持续加强央企、省企、民企、外企对接，对重大招商项目实行“一对一”全程跟踪服务，力争厦钨稀土分离项目、机械科学研究总院海西分院高端装备产业园二期、生物医药产业园、石墨（烯）产业孵化园、尤溪经编染整产业园等一批重大产业项目加快落地。

　　（三）统筹城乡协调发展，推进美丽三明建设。城乡是我家，美丽靠大家。要牢固树立“绿水青山就是金山银山”理念，加快新型城镇化建设，大力实施乡村振兴战略，努力实现生态环境“高颜值”和经济发展“高素质”。

　　推进县域经济发展。坚持以城促产、强产兴城、产城融合，支持永安、沙县发挥引领带动作用，持续争创全省县域经济实力“十强”县；支持尤溪、大田、将乐不断壮大总量、提升质量，争创全省县域经济发展“十佳”县；支持明溪、清流、宁化、建宁、泰宁加快脱贫步伐，发展壮大特色产业，做大县域特色经济。深入实施“大城关”战略，抓好县城规划和建设，有序推进新区开发和旧城改造，推动县城向区域中小城市提升。深化三明国家中小城市综合改革试点和尤溪洋中镇省级“小城市”培育试点，推进永安国家级、大田省级新型城镇化试点建设，加快基本公共服务向常住人口覆盖，引导人口向城市和建制镇集聚。

　　实施乡村振兴战略。坚持农业农村优先发展，按照产业兴旺、生态宜居、乡风文明、治理有效、生活富裕的总要求，建立健全城乡融合发展机制和政策体系，加快推进农业农村现代化。鼓励乡村立足资源优势，因地制宜发展“一乡一业”“一村一品”，加快5个省级特色小镇创建工作，培育一批产业特色鲜明的工业小镇、农业小镇、森林小镇、文旅小镇，推动乡村优势产业集聚发展。完善农村水电讯等基础设施，加快推进“四好农村路”建设，实行“路长制”，改扩建农村公路300公里以上。加强农村人居环境整治，深入实施“千村整治、百村示范”工程，注重历史文化资源和传统村落保护与传承，争创一批国家、省级历史文化名镇名村和传统村落，支持老区村、少数民族乡村加快发展。大力培育新型农民，支持农民就业创业，促进农民增收。健全自治、法治、德治相结合的乡村治理体系，做好村（居）委会换届工作，培养造就一支懂农业、爱农村、爱农民的“三农”工作队伍。

　　打好污染防治攻坚战。强化大气环境整治，突出抓好三钢节能减排系统改造工程，严格三钢大气污染物排放限值，提升市区空气质量；开展臭氧污染专项治理，加强建筑施工扬尘防治。强化水环境整治，狠抓工业污染、畜禽养殖污染、涉砂行为、城乡生活污水垃圾、小水电生态等五个方面专项治理，力争年底前消灭Ⅲ类以下小流域；落实水源保护区管护责任，依法保护东牙溪和薯沙溪水库饮用水源。强化土壤污染防治，完成农用地污染状况详查工作，启动土壤污染治理与修复示范区建设，开展矿山地质环境治理恢复专项整治。强化生态系统保护，实施闽江流域山水林田湖草生态保护修复工程试点项目，综合治理水土流失37万亩，造林绿化和森林经营195万亩。

　　（四）突出发展市区经济，加快生态新城建设。中心城市是发展龙头，三明发展好不好，要靠龙头引着跑。要进一步做大做强中心城市，深入实施市区经济重点项目建设行动计划，加快打造现代山水名城，不断提升三明中心城市经济实力和辐射带动力。

　　提升市区产业发展水平。要推动市区工业强起来，持续深化市区九个专业特色工业园区市领导挂包工作机制，加快园区基础设施建设，力争引进投资亿元以上项目10个；实施市区工业重点项目50个，抓好三钢17个转型升级项目、三化氟化工项目、厦钨三元正极材料项目、省电子信息集团智能化车载模组项目等大项目建设。要推动市区服务业旺起来，加快北部新城、南部新城开发，以万达广场、徐碧商务区、东霞—台江核心区等项目为龙头，推动休闲娱乐、电子商务、现代物流、总部经济等服务业集聚发展，提升主城区商贸服务品质；加快三明郊野国家地质公园开发建设，重点打造“瑞云山—碧溪—大佑山”和“百年栲、千年寨、万年居”2条休闲旅游带，建成三明南旅游集散服务中心。要推动市区农业特起来，依托市区优势率先发展互联网定制农业、众筹农业、智慧农业等新模式，抓好陈大十里休闲农业观光带、清枫谷生态农业观光园、中村兰花基地等项目建设，着力打造城郊型现代农业体系。

　　提升市区城市规划建设管理水平。要推动市区美起来，大力推进“城市双修”全国试点城市建设，着力打造现代山水名城。持续抓好三明绿道连接线工程、地灾隐患点整治工程、新市路道路提升和交通改善工程、公共自行车道工程、公共停车设施建设工程、贵溪洋生态湿地公园等20个示范项目建设，加快解决出行难、停车难、水土流失等问题，完善社区公共卫生、便民服务、居家养老等基础设施建设，进一步优化城市功能。扎实推进城市执法体制改革，强化城市管理数字化、精细化、规范化，持续整治背街小巷、餐厨油烟和渣土车“跑冒滴漏”等突出问题，展示全国文明城市新风貌、新形象。

　　提升生态新城开发水平。深入推进三明生态工贸区建设，继续以“十同”为重点，加快市区与沙县、永安同城化步伐。生态新城是市区经济的重要组成部分，要突出抓好生态新城“一带六区”建设，加快铁路冷链物流特色小镇、医养特色小镇的规划设计，推进水南污水处理厂、职教园二期等公共服务配套建设；大力发展冷链物流、旅游养生、文化创意、电子商务等现代服务业，加快清华启迪项目建设，积极争创国家（三明）全民健康科技创新先行区，提升产业集聚发展水平。

　　（五）全面深化改革开放，增创体制机制优势。没有改革就没有动力，没有开放就没有活力。要持续全面深化改革，积极参与“一带一路”建设，用改革开放的办法为发展增添新动能。

　　全面深化重点改革。持续深化医改，以全民健康为中心，完善总医院（医联体）运行机制，全面实行按疾病诊断相关分组收付费改革，落实分级诊疗和家庭医生签约服务。持续深化林改，强化林业金融创新，促进林权规范流转和林地规模经营，积极推进林业PPP建设项目，加快林业资产资本化进程，力争全市新型林业经营组织覆盖面达60%以上。深化农村改革，全面完成农村土地承包经营权确权登记颁证，完善农村承包地“三权分置”改革，全面推进农村集体产权制度改革。加快国有企业改革发展步伐，全面实行公司制改制，推进市属国有企业资产重组和企业集团组建。深化预算制度改革，开展财政专项资金清理整合，加强政府性债务管理。全面完成国家生态文明试验区改革任务，开展领导干部自然资源资产离任审计，统筹推进三明市国家低碳城市试点、三明生态新城国家低碳城（镇）试点建设，巩固提升“河长制”，推行“湖长制”。深化基础教育“总校制”改革，落实“1+4”正向激励机制，推行中小学教师“县管校聘”，完善名师名校长遴选培育机制，全力办好人民满意的教育。

　　积极扩大对外开放。主动融入海上丝绸之路核心区，重点发展壮大16家年出口额超千万美元的生产型企业，扩大食品、化工产品、竹木制品、纺织品等特色优势产品出口规模，推动钢铁、机械装备、汽车、水泥等优势生产企业“走出去”。主动承接自贸区“外溢”效应，提升“陆地港”跨关区电子关锁服务功能，提升保税仓库运营水平，吸引自贸区企业来明投资或开展业务。进一步拓展对外交流交往，深化明台交流合作，密切与港澳地区、国外友城的沟通联系，加强与海外华侨华人及其社团的友好往来。

　　（六）着力发展社会事业，不断增进民生福祉。人民对美好生活的向往就是我们的奋斗目标，人民想什么，我们就要干什么。要坚持尽力而为、量力而行，努力在幼有所育、学有所教、劳有所得、病有所医、老有所养、住有所居、弱有所扶上取得新成效，不断增强群众获得感、幸福感、安全感。

　　打好精准脱贫攻坚战。深化国家扶贫改革试验区建设，提升“348”精准扶贫机制，确保建档立卡贫困人口全部脱贫，11个市级扶贫开发工作重点乡、2个省级扶贫开发工作重点县摘帽，完成造福工程易地扶贫搬迁2700人，并对已摘帽的县、乡、村、户，在帮扶机制、帮扶措施上力度不减、政策不变，确保实现稳定脱贫。总结推广市区“城市困难家庭精准帮扶试点”经验，进一步改善城市困难家庭生活条件。

　　加快补齐民生短板。突出教育、医疗卫生、养老和城乡民生基础设施四个领域，全力推进补短板项目建设。着力解决“择校热”“大班额”等突出问题，实施中小学扩容工程20个，新建、改扩建公办幼儿园项目18个，分别新增学位5600个、6300个；有序推进市区教育布局调整，加快推进附小扩容改造等项目建设，打造市区优质教育高地；支持三明学院通过教育部本科教学工作审核评估，完善三明医学科技职业学院托管职教园区工作机制，推动职业教育产教融合，支持特殊教育、终身教育、民办教育、老年教育发展。实施医学三大优秀人才培养计划，加快推进儿科、产科、精神科等薄弱学科和消化内科、眼科、骨科等重点学科建设，扶持中医药事业发展，完善妇幼保健体系，创新计划生育服务管理，加快乡村卫生院（所）标准化建设。推进国家级医养结合试点城市、省级养老服务试点示范建设，加快构建以居家为基础、社区为依托、机构为补充的养老服务体系，新建农村幸福院55所、社区日间照料中心10所。统筹实施交通畅通、防洪防涝、管网建设、智慧城市等九大工程，新建、改造城市道路和各类市政管网500公里以上，新增城乡公共停车位2500个，新建改建城乡公共厕所256座。

　　推动文化繁荣发展。培育和践行社会主义核心价值观，深入开展群众性精神文明创建活动，加强未成年人思想道德教育，抓好家庭家教家风建设，推进诚信建设和志愿服务制度化，倡导移风易俗，树立良好风尚。巩固提升国家公共文化服务体系示范区创建成果，加快国家级客家文化（闽西）生态保护实验区建设，推进红色文化、朱子文化（闽学文化）传承发展，鼓励文艺创作，加强文物和文化遗产保护利用。健全现代文化产业体系和市场体系，大力发展文化创意、文化旅游等新型业态。深入实施全民健身计划，完善城市社区和农村全民健身设施，重视老年人体育事业，积极备战第十六届省运会和第十届省老健会，承办好省第九届少数民族传统体育运动会。

　　完善社会保障体系。加强公共就业服务，精准帮扶高校毕业生、就业困难人员、退役军人等重点群体就业，新增城镇就业1.8万人以上。建立健全重大群体讨薪事件防控机制、劳动人事争议调解仲裁机制，构建和谐劳动关系。加大社会保险扩面力度，实施全民参保计划。提高困难群众补助水平，加快推进城乡低保标准一体化。加强困难群众住房保障，实施棚户区改造933套、基本建成405套。健全未成年人、农村留守儿童、妇女、残疾人、失独家庭关爱服务体系。发展民族宗教、社会福利、社会救助、红十字会、慈善、老龄事业，支持关心下一代工作。

　　加强和创新社会治理。深入开展“七五”普法，持续抓好“法律六进”工作。深化“平安三明”建设，完善“雪亮工程”，加大对涉黑涉恶、涉赌涉毒案件和网上虚假信息诈骗等打击整治力度，保障群众生命财产安全。健全社区治理体系，不断提升社区治理法治化、科学化水平。依法依规开展信访工作，努力解决群众合法合理诉求。强化食品药品监管，深化治理“餐桌污染”，全面推进食品安全“一品一码”全过程追溯体系建设。推进安全生产领域改革发展，深化重点行业领域专项整治，坚决遏制重特大安全生产事故发生。加强应急处置能力建设，提高防灾减灾救灾水平。密切与各民主党派、工商联、无党派代表人士的联系与协商，支持工会、共青团、妇联等群团组织发挥更大作用。推进双拥共建，加强国防动员和人民防空建设，深化军民融合发展。

　　三、全力建设务实高效创新廉洁的服务型政府

　　新时代、新目标、新任务，政府要有新气象、新担当、新作为。我们要深入学习贯彻党的十九大精神，牢固树立“四个意识”，严守政治纪律和政治规矩，始终践行全心全意为人民服务的根本宗旨，大力倡导“务实、高效、创新、廉洁”作风，不断提高政治能力、执行能力、创新能力、服务能力。

　　（一）旗帜鲜明讲政治。把政治建设摆在首位，以更深的感情、更自觉的行动，坚决维护习近平总书记在党中央、在全党的核心地位，坚决维护以习近平同志为核心的党中央权威和集中统一领导，切实在思想上政治上行动上同以习近平同志为核心的党中央保持高度一致。强化思想理论武装，深刻学习领会习近平新时代中国特色社会主义思想，推进“两学一做”学习教育常态化制度化，扎实开展“不忘初心、牢记使命”主题教育，切实用党的创新理论武装头脑。提高执行能力，坚决贯彻落实党中央国务院和省委省政府、市委决策部署，决不允许各行其是、各自为政，决不允许有令不行、有禁不止，决不允许搞上有政策、下有对策，切实做到“不打任何折扣，不耍任何小聪明，不搞任何小动作”。

　　（二）务实创新求实效。更加注重务实，坚持一线工作法，能在现场就不在会场，深入基层调查研究，积极开展现场办公，推动部门在一线服务、问题在一线解决、干部在一线锻炼。更加注重高效，弘扬“马上就办、真抓实干”精神，坚决整治“为官不为”“庸懒散拖”行为，深化“放管服”改革，拓展“一窗式”并联审批领域，落实“一趟不用跑”和“最多跑一趟”办事清单，加快“互联网+政务服务”建设，推进全流程网上办理。更加注重创新，积极践行新发展理念，持续推进重点领域机制创新，健全完善正向激励、容错纠错机制，旗帜鲜明为敢于担当的干部担当，为敢于负责的干部负责，鼓励干部主动想、自主改、大胆试，争当机制创新的“策源地”。

　　（三）清正廉洁守底线。尊崇和维护宪法，用法治思维和法治方式想问题、作决策、办事情，加强立法工作和规范性文件备案审查，提升行政执法水平，加快建设法治政府。始终坚持公开透明，自觉接受人大及其常委会法律监督和工作监督、政协民主监督，充分尊重司法监督，主动接受公众舆论监督，加强层级监督和审计监督，让权力在阳光下运行。深入推进党风廉政建设和反腐败斗争，巩固拓展落实中央八项规定精神成果，坚决防止“四风”问题反弹回潮。进一步扎紧制度笼子，完善土地招拍挂、工程招投标、国有资产处置、扶贫资金监管等制度，保证干部清正、政府清廉、政治清明。

　　各位代表，新时代鼓舞人心，新蓝图催人奋进！让我们更加紧密地团结在以习近平同志为核心的党中央周围，认真践行习近平新时代中国特色社会主义思想，在市委的坚强领导下，以永不懈怠的精神状态和一往无前的进取姿态，拿出勇气，拿出干劲，为“再上新台阶、建设新三明”而努力奋斗！
